# Buffalo Medical and Surgical Association

**Published:** 1891-07

**Authors:** W. S. Renner

**Affiliations:** Secretary


### BUFFALO MEDICAL AND SURGICAL ASSOCIATION.

Reported by W. S. RENNER, M. D., Secretary.

Regular meeting, held May 5, 1891.

The President, Dr. W. C. Phelps, in the chair.

Dr. John Parmenter read a paper entitled, Subclavicular Dislocation of the Humerus — Open Arthrotomy — Recovery. Vide page 705 this Journal.

DISCUSSION.

Dr. W. C. Phelps had never seen open arthrotomy performed for this condition; he had never gone beyond extension (pulleys etc). But few cases of the operation had been reported, because the indications for the operation seldom arise. He had once reduced a subclavicular dislocation of the humerus by placing the patient on his back and extending both arms above his head, while he kept his (the operator's) feet on the patient's shoulders; considerable paralysis of the deltoid followed the reduction in this case, and was still present when the patient was last seen, a year and a half after the operation.

Dr. Hayd asked Dr. Phelps if he had used chloroform to reduce the dislocation of which he had spoken.

Dr. Phelps replied that he always employed chloroform to reduce dislocations.

Dr. Parmenter claimed that the cases of which he had spoken in his paper, could not be reduced by any other method.

REPORTS OF PREVAILING DISEASES.

Dr. Rochester had seen three cases of membranous tonsillitis, which he did not consider diphtheritic; temperature, 100; membrane disappeared under mild local treatment.

Dr. Bartlett spoke of the masking effect of La Grippe on other diseases. He described a condition which he styled "Grippe tongue." He had seen no diphtheria and very little croup during the past Winter.

Dr. Goltra had also seen some cases of membranous tonsillitis; he did not consider the disease contagious; he had also seen what was to him a peculiar condition of the conjunctiva, characterized by hyperemia, and peculiar spots; it seemed to be contagious. He had never seen the condition before; it disappeared under mild treatment.

Dr. Grant had had under his care several cases of the disease of the eye, of which Dr. Goltra had spoken ; it is known as pink-eye, or acute conjunctival catarrh ; he treated his cases with mild astringents and antiseptics. The spots of which Dr. Goltra had spoken were probably marginal phlyctenulæ.

Dr. Parmenter had also noted the rarity of diphtheria during the past Winter.

Dr. E. H. Long asked Dr. Rochester how he distinguished the various membranous diseases from each other.

Dr. Rochester stated that he distinguished the form of which he had spoken from diphtheria, by the appearance of the membrane and the slight amount of constitutional disturbance. He had seen considerable spasmodic croup during the past Winter, but no diphtheria ; he had also seen considerable congestive nephritis in connection with Grippe.

Dr. Hayd had found little good resulting from administering medicine in Grippe, especially in the bronchitis of that disease ; had found the best results followed keeping the patient in the house and administering small doses of codeia.

Dr. Rochester had noted the same peculiarity as Dr. Hayd ; in these cases he had found albumin in the urine ; such cases he had placed on distilled or Bethesda water, and insisted on these remaining in the house until their tongues were perfectly clean.

Dr. F. H. Potter had found that the sequelæ of Grippe were usually one of the following two conditions, viz., otitis media or laryngitis, extending to the trachea and lower bronchi, accompanied by considerable cough. He held that the membranous conditions found in the throat were more or less allied.

Dr. Parmenter had reasons for having great confidence in ten gr. doses of oxalate of cerium as a remedy in chronic dry coughs, and he thought it might be beneficial in the cases of which Dr. Hayd had spoken.

Dr. Phelps had recently seen a case of simple follicular tonsillitis, with no fever, which was reported to the Board of Health as a case of diphtheria.

---

Koch on His Muscle.—It is announced that Professor Koch is preparing a reply to all the criticisms that have been made on his method. Professor Virchow's objections are to be dealt with in detail.—*St. Louis Med. and Surg. Journal.* Perhaps Professor Senn will also receive some attention.

